# Comparison of oncologic and functional outcomes between radical nephroureterectomy and segmental ureterectomy for upper urinary tract urothelial carcinoma

**DOI:** 10.1038/s41598-021-87573-5

**Published:** 2021-04-09

**Authors:** Tae Heon Kim, Chung Un Lee, Minyong Kang, Hwang Gyun Jeon, Byong Chang Jeong, Seong Il Seo, Seong Soo Jeon, Hyun Moo Lee, Hyun Hwan Sung

**Affiliations:** 1 Department of Urology, CHA Bundang Medical Center, CHA University, Seongnam, Republic of Korea; 2 Department of Urology, Samsung Medical Center, Sungkyunkwan University School of Medicine, 81, Irwon-ro, Gangnam-gu, Seoul, 06351 Republic of Korea

**Keywords:** Cancer, Oncology, Urology

## Abstract

This study aims to compare oncologic and functional outcomes after radical nephroureterectomy (RNU) and segmental ureterectomy (SU) in patients with upper urinary tract urothelial carcinoma (UTUC). We retrospectively collected data on patients who underwent either RNU or SU of UTUC. Propensity score matching was performed among 394 cases to yield a final cohort of 40 RNU and 40 SU cases. Kaplan–Meier analysis and the log-rank test were used to compare overall survival (OS), cancer-specific survival (CSS), progression-free survival (PFS), and intravesical recurrence-free survival (IVRFS) between the groups. We also compared the change in postoperative estimated glomerular filtration rate (eGFR). There was no significant difference in terms of CSS, PFS, and IVRFS between the RNU and SU groups, but the RNU group had a better OS than the SU group (p = 0.032). Postoperative eGFR was better preserved in the SU group than in the RNU group (p < 0.001). SU provides comparable CSS, PFS, and IVRFS for patients with UTUC compared to RNU, even in patients with advanced-stage and/or high-grade cancer. Further, SU achieves better preservation of renal function.

## Introduction

Upper urinary tract urothelial carcinoma (UTUC) is a relatively uncommon disease, accounting for 5–10% of urothelial carcinomas overall, and with an estimated annual incidence of roughly two people per 100,000^1,2^. Of all UTUCs, approximately 25% arise from the ureter, while the remaining 75% occur in the collecting system of the kidney^3^. Although radical nephroureterectomy (RNU) with bladder cuff excision has been the gold-standard for management of non-metastatic UTUC, the resultant solitary kidney status may lead to higher rates of surgically induced renal insufficiency, dialysis, cardiovascular morbidity, and overall mortality^4–8^.

Given the RNU-related morbidity, contemporary treatment strategies for non-metastatic UTUC aim to reduce the morbidity associated with RNU while optimizing oncologic outcomes in appropriately selected patients. In response, kidney-sparing surgery (KSS) is currently being used as an alternative treatment for UTUC because it allows for a less invasive procedure and preserves the ipsilateral kidney. KSS options depend on tumor focality, size, grade, and location and include endoscopic ablation or resection, percutaneous management, and segmental ureterectomy (SU). According to current management guidelines for UTUC, SU could be an option even in patients with high-risk UTUC located in the ureter^2^. SU can provide adequate pathological specimens for staging and grading and can be performed with lymphadenectomy^2,9^. However, despite the putative advantages of SU over RNU, little is known about whether SU contributes to better preservation of renal function while achieving acceptable oncologic outcomes. To date, retrospective data concerning SU for UTUC are conflicting and somewhat inconclusive with respect to renal functional advantage in comparison with RNU^10–14^.

Due to the lack of high-quality randomized studies focused on oncologic and functional outcomes of SU, we aimed to clarify the potential therapeutic benefit of SU. To address this unmet issue, we compared oncologic control and renal function recovery of patients that underwent SU and RNU.

## Materials and methods

### Study population and outcome parameters

We collected the data from 465 consecutive patients who were treated with RNU (n = 418) and SU (n = 47) for UTUC between January 2008 and December 2016 at our institution. Among these patients, 24 patients in RNU group and three patients in SU group were excluded for the following reasons: a bilateral UTUC (n = 3 in RNU group and n = 1 in SU group); a history of previous UTUC (n = 2 in RNU group and n = 1 in SU group); previous or concomitant radical cystectomy due to invasive bladder cancer (n = 9 in RNU group); other malignancies (n = 13 in RNU group and n = 2 in SU group); a history of neoadjuvant chemotherapy (n = 2 in RNU group); there was some patient overlap. The remaining 438 patients were further analyzed.

The retrospective study was conducted in accordance with guidelines from the Declaration of Helsinki and the STROBE statement^15^. This study was approved by our institutional review board (IRB) of Samsung Medical Center (IRB approval number: 2020-06-54). The IRB waived the requirement for informed consent from the included patients due to the retrospective nature of this study. Clinicopathologic characteristics and oncologic outcomes were recorded. Preoperative and postoperative estimated glomerular filtration rate (eGFR) data were also collected. eGFR was calculated from serum creatinine using the Modification of Diet in Renal Disease formula, which adjusts for age and sex^16^.

### Surgical procedures

The choice of either RNU or SU were mainly determined depending on the surgeon’s preference and the patient's informed consent after counseling regarding the surgical methods according to tumor location. For RNU, the kidney and the ureter with a bladder cuff were removed en bloc. For SU, surgical approaches depend on tumor location. For distal tumors, segmental resection of distal ureter with a bladder cuff excision and ureteral re-implantation were performed. Ureteroneocystostomy or a psoas bladder hitch was used as re-implantation. For mid or proximal tumors, ureterectomy including the distal ureter with a bladder cuff excision was mostly carried out. When ureteral length is insufficient for remnant ureter re-implantation, partial ureterectomy with end to end ureteric anastomosis, or an ileal ureter or ureterocutaneostomy was used. Indication and the extent of lymphadenectomy were usually determined by the surgeon’s discretion based on preoperative image work-up, tumor location, and patient characteristics.

### Patient follow-up

The patients were generally followed up every 3–4 months for the first 2 years after surgery, every 6 months from third to fifth years, and annually thereafter. For patients who underwent SU, ureteroscopy was usually performed every 6 months, based on the surgeon’s discretion. Intravesical recurrence-free survival (IVRFS) was defined as the time between surgery and the date of tumor recurrence in the bladder. Progression-free survival (PFS) was defined as the time from surgery to either local tumor relapse in previous surgical area or distant metastasis. Cancer-specific survival (CSS) and overall survival (OS) were defined as the time between surgery and death from UTUC and from any cause, respectively.

### Statistical analyses

Descriptive statistics included frequencies and proportions for categorical variables. Continuous variables were presented as means ± standard deviations for normal distributed data and median (interquartile ranges [IQRs]) for non-normally distributed data. Categorical variables were compared using either Pearson’s chi-square test or the stratified chi-square test. Fisher’s exact test was also used when appropriate. The Shapiro–Wilk normality test was examined to check normal distribution in continuous variables, followed by Student’s *t*-test to assess differences between variables. For variables with a non-normal distribution, differences between variables were assessed using the Mann–Whitney U-test. To account for potential important differences between the two treatment groups, patients who underwent either RNU or SU were matched 1:1 with propensity scores via the nearest neighbor matching algorithm without replacement^17^. Thus, the propensity score-matched cohort was balanced according to patient’s characteristics, namely, age at surgery, sex, year of surgery, pathological T stage, and tumor grade.

The primary outcome of this study was to compare RNU and SU in terms of OS, CSS, PFS, and IVRFS. Thus, the Kaplan–Meier method was used to assess OS, CSS, PFS, and IVRFS of the two treatment groups in the entire study cohort, advanced stage subgroup (pT2-4), and high-grade subgroup (grade 3). The log-rank test was used to assess differences. As a secondary outcome, we analyzed renal function, which was usually assessed by eGFR. Postoperative change and preservation rate of eGFR were compared between the RNU and SU groups at both 1 and 12 months. The eGFR preservation rate was calculated as preoperative eGFR value divided by postoperative eGFR value. Statistical analysis was performed using the SAS software, version 9.4 (SAS Institute Inc., Cary, NC, USA). All tests were two-sided, with p < 0.05 considered to indicate a statistical significance.

## Results

After excluding 27 patients, we finally analyzed 394 patients underwent RNU, and 44 patients were treated with SU during the study period. Patients’ clinicopathologic characteristics are summarized in Table 1. After propensity score matching, a total of 40 patients were available for analysis in each cohort. Overall, the median age at surgery was 71 years (IQR 61–74 years), and two-thirds of the patients (55/80) were male. Median follow-up duration was 23.2 months (IQR 10.8–33.0 months). There were no significant differences between the two groups, except for history of previous bladder cancer, approach type of surgery, tumor length, and follow-up duration. Relative to the RNU group, the SU group had a previous history of bladder cancer more frequently (p < 0.001), underwent the open surgery approach more often (p < 0.001), and had a shorter tumor length (p = 0.005) and follow-up duration (p < 0.001). There was a significant difference in tumor location between the two groups (p < 0.001). In the RNU group, 18 (45%) and 22 (55%) patients had UTUC localized in the ureter and renal pelvis, respectively. Meanwhile, all 40 patients in the SU group had UTUC localized in the ureter. Among the patients in the SU group, 28 (70%) patients had elective indications with normal contralateral kidney and the remaining 12 (30%) patients had imperative indication. After SU with bladder cuff excision, re-implantation was performed as follows: ureteroneocystostomy (33 patients, 82.5%); ileal ureter (two patients, 5.0%); psoas bladder hitch (one patient, 2.5%); ureterocutaneostomy (one patient, 2.5%). Three patients (7.5%) underwent partial ureterectomy with end to end ureteric anastomosis.

**Table 1:** Comparison of clinicopathological characteristics between the RNU and SU groups in the original and matched data sets.

	Before propensity score matching			After propensity score matching		
	RNU group	SU group	*p* value	RNU group	SU group	*p* value
No of patients	394	44		40	40	
Age (years)	66.0 (57.0, 74.0)	69.5 (61.0, 74.5)	0.173	71.0 (61.0, 74.5)	69.5 (61.0, 74.0)	0.795
Sex, male	300 (76.1)	30 (68.2)	0.245	28 (70.0)	27 (67.5)	0.809
**Year of surgery**			< 0.001			0.390
2008–2010	102 (25.9)	3 (6.8)		6 (15.0)	3 (7.5)	
2011–2014	172 (43.7)	6 (13.6)		3 (7.5)	6 (15.0)	
2015–2016	120 (30.5)	35 (76.6)		31 (77.5)	31 (77.5)	
History of previous bladder cancer	88 (22.3)	19 (43.2)	< 0.001	9 (22.5)	17 (42.5)	< 0.001
**Approach type of surgery**			< 0.001			< 0.001
Open	213 (54.1)	36 (81.8)		21 (52.5)	34 (85.0)	
Laparoscopic	181 (45.9)	1 (2.3)		19 (47.5)	1 (2.5)	
Robot-assisted	0 (0)	7 (15.9)		0 (0.0)	5 (12.5)	
Tumor length (mm)	32.0 (22.0, 45.0)	20.0 (13.5, 25.5)	< 0.001	27.0 (20.0, 40.0)	20.5 (14.5, 27.0)	0.005
**Pathological T stage**			0.157			0.714
Tis	9 (2.3)	1 (2.3)		2 (5.0)	1 (2.5)	
Ta	45 (11.4)	8 (18.2)		9 (22.5)	5 (12.5)	
T1	112 (28.4)	10 (22.7)		10 (25.0)	10 (25.0)	
T2	73 (18.5)	14 (31.8)		11 (27.5)	13 (32.5)	
T3	148 (37.6)	11 (25.0)		8 (20.0)	11 (27.5)	
T4	7 (1.8)	0 (0)		0 (0)	0 (0)	
**Pathological N stage**			0.347			0.556
Nx/No	364 (92.4)	43 (97.7)		38 (95.0)	39 (97.5)	
≥ N1	30 (7.6)	1 (2.3)		2 (5.0)	1 (2.5)	
**Tumor grade**			0.017			0.271
1	12 (3.1)	5 (11.4)		3 (7.5)	2 (5.0)	
2	177 (44.9)	15 (34.1)		21 (52.5)	15 (37.5)	
3	205 (52.0)	24 (54.6)		16 (40.0)	23 (57.5)	
Concomitant LVI	75 (19.0)	6 (13.6)	0.382	6 (15.0)	6 (15.0)	> 0.999
Adjuvant chemotherapy	95 (24.1)	8 (18.2)	0.379	7 (17.5)	8 (20.0)	0.775
Follow-up duration (months)	39.5 (24.7, 69.5)	11.2 (3.8, 23.1)	< 0.001	28.2 (22.9, 37.8)	11.2 (5.2, 23.5)	< 0.001

Values are presented as number (%). The Shapiro–Wilk normality test was used to investigate the normal distribution of continuous variables. Continuous and normally distributed variables are presented as mean ± standard deviations, and continuous and non-normally distributed variables are presented as medians with interquartile ranges.

*RNU* radical nephroureterectomy, *SU* segmental ureterectomy, *ASA* American Society of Anesthesiologists, *LVI* lymphovascular invasion.

**Figure 1:**
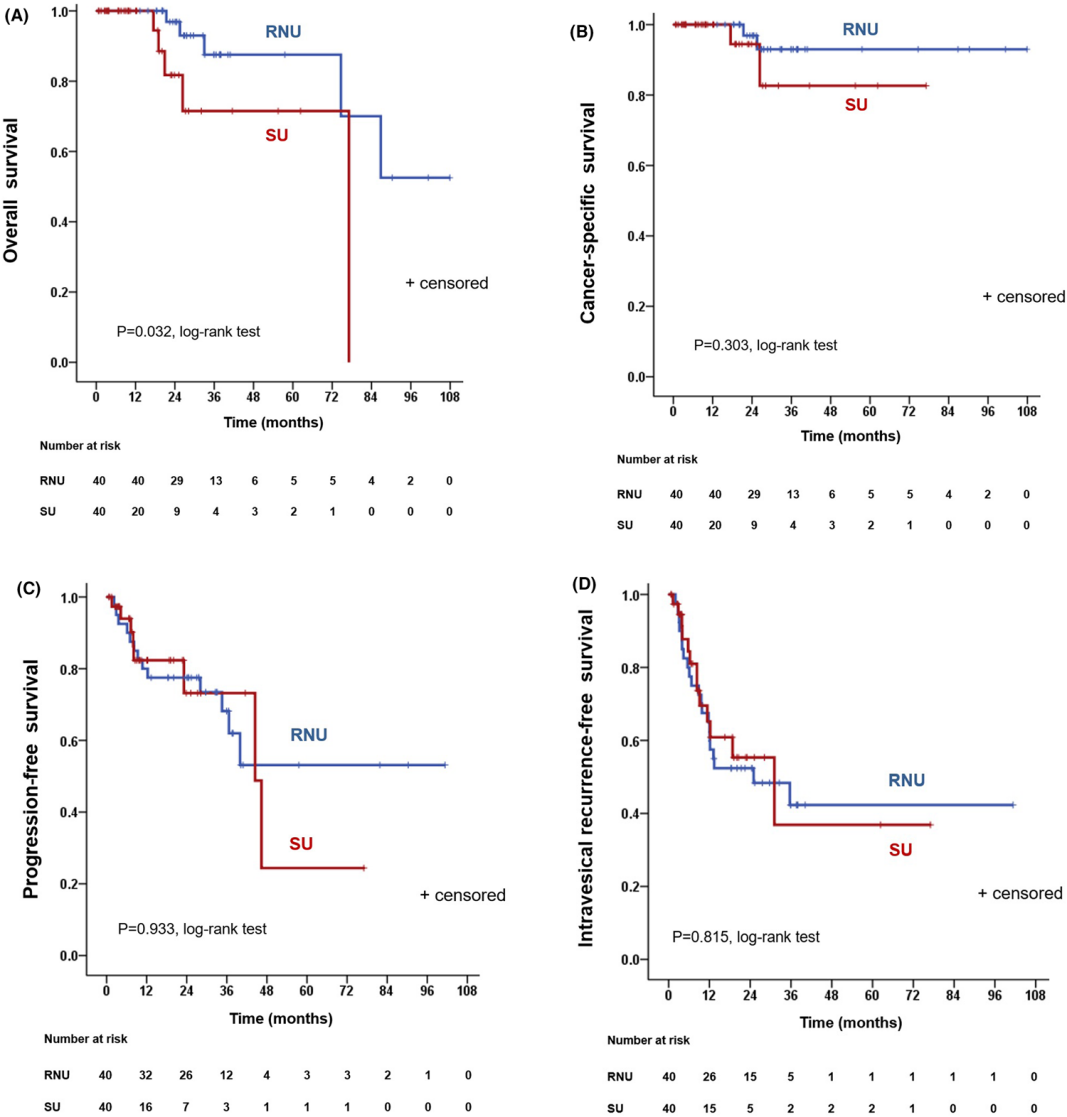
In the entire cohort, Kaplan–Meier analysis depicting (**A**) overall survival; (**B**) cancer-specific survival; (**C**) progression-free survival; (**D**) intravesical recurrence-free survival for patients who underwent RNU or SU, after 1:1 propensity score matching. *RNU* radical nephroureterectomy, *SU* segmental ureterectomy.

Ten patients died (5 RNU group vs 5 SU group) during the follow-up. The 3-year OS estimates were 87.5% and 71.5% for the RUN and SU group, respectively, indicating that the RNU group had a better 3-year OS than the SU group (p = 0.032, Fig. 1A). The 3-year CSS estimates were 93.0% and 82.6% for the RUN and SU group, respectively, which was not significantly different (p = 0.303, Fig. 1B). There were no statistically significant differences in terms of 3-year PFS and IVRFS estimates among patients treated with RNU and SU (68.2% vs 73.2%, p = 0.933, Fig. 1C; 42.3% vs 36.9%, p = 0.815, Fig. 1D; respectively). Figure S1 shows survival curves in patients with advanced stage disease (pT2-4). Among patients with advanced stage disease, the RNU group had a significantly better OS than the SU group (p = 0.015; Fig. S1A). However, CSS, PFS, and IVRFS were similar for the RUN and SU groups in those with advanced stage disease (p = 0.167, Fig. S1B; p = 0.639, Fig. S1C; p = 0.912, Fig. S1D; respectively). When patients were stratified by grade, there were no significant differences between the RNU and SU group in terms of OS, CSS, PFS, and IVRFS in high-grade patients (grade 3) (Fig. S2).

Table 2 shows changes in eGFR in the RNU and SU groups. There was no significant difference in preoperative eGFR among patients treated with RNU and SU. Postoperatively, eGFR in the SU group was significantly improved compared with the RNU group at both 1 month and 1 year. Patients treated with SU had significantly better preservation of renal function than the those treated with RNU both 1 month and 1 year postoperatively (all p < 0.001).

**Table 2:** Comparison of preoperative and postoperative eGFRs between the two groups.

	RNU group (n = 40)	SU group (n = 40)	*p* value
Preoperative eGFR	65.5 ± 17.7	62.2 ± 21.4	
**Postoperative 1-month eGFR**	49.6 ± 11.4	65.3 ± 21.0	
Change in eGFR^a^	− 16.0 ± 16.1	3.8 ± 10.1	< 0.001
eGFR preservation rate^a^ (%)	78.8 ± 18.9	108.5 ± 20.0	< 0.001
**Postoperative 12-month**	49.7 ± 12.9	65.4 ± 22.1^b^	
Change in eGFR^a^	− 15.8 ± 17.8	3.4 ± 16.7	< 0.001
eGFR preservation rate^a^ (%)	79.6 ± 23.7	110 ± 30.7	< 0.001

The Shapiro–Wilk normality test was used to investigate the normal distribution of continuous variables. Continuous and normally distributed variables are presented as mean ± standard deviations.

eGFR is expressed in mL/min/1.73 m^2^.

*eGFR* estimated glomerular filtration rate, *SU* segmental ureterectomy, *RNU* radical nephroureterectomy.

^a^Versus preoperative eGFR.

^b^Postoperative eGFR values at 12 months were available for 34 (85%) patients who underwent SU.

## Discussion

In this propensity matched comparison, we found that SU and RNU offer equivalent 3-year CSS, PFS, and IVRFS in patients with non-metastatic UTUC, and that renal function was better preserved in patients treated with SU compared with RNU. Of note, in a subgroup analysis by tumor stage or tumor grade, there were no significant differences between SU and RNU in terms of 3-year CSS, PFS, and IVRFS for advanced stage or high-grade patients. These findings imply that SU may be useful in well selected patients with high-grade or high stage UTUC, although previous reports have shown that patients with low-grade, low-stage UTUC have been successfully managed with KSS such as endoscopic or percutaneous management^18,19^.

In this study, RNU significantly improved OS, with 3-year OS estimates of 87.5% for RNU and 71.5% for SU. However, this result should be interpreted with caution. Poor OS in the SU group could be related to the fact that some patients (30%, 12/40) had imperative indications, such as chronic renal insufficiency or solitary kidney. Thus, in this group, comorbidities might be a potential confounder for oncologic outcomes. Previous studies have shown that preoperative renal insufficiency is an independent predictor of poor OS in patients with UTUC^20,21^. Indeed, in our study, four of five patients who died during the follow-up period in the SU group had imperative indications. Moreover, a relatively small number of enrolled patients and short-term follow-up duration may affect differences in OS.

Initial experience of KSS for UTUC has been limited to patients with solitary kidney, bilateral disease, or severe renal insufficiency. However, growing experience with KSS, coupled with improved technology and appropriate risk stratification, may lead to widespread expansion of KSS to patients with a normal contralateral kidney. A number of studies, though limited to retrospective, unmatched comparative studies, have reported no short and mid-term difference in oncologic outcomes between RNU and KSS in patients with UTUC^9,18,19^. Regarding oncologic outcomes, our results are in concordance with previous studies that have reported that SU have comparable oncologic outcomes to RNU. In a retrospective study of 26 patients with UTUC who underwent SU (n = 12) and RNU (n = 14), Kato et al.^22^ reported that the 5-year CSS (87.5% vs. 71.9%; p = 0.69) and recurrence-free survival (34.4% vs. 50.0%; p = 0.95) appeared to be comparable between SU and RNU. A meta-analysis of 11 publications by Fang et al.^23^ also demonstrated no significant differences between SU and RNU in terms of OS (hazard ratio 0.90; p = 0.33), CSS (hazard ratio 0.98; p = 0.93), and recurrence-free survival (hazard ratio 1.06; p = 0.72). In their analysis, the reported 5-year cumulative OS, CSS, and recurrence-free survival in the SU group ranged from 40 to 72%, 54–90%, and 24–84%, respectively. While the reported 5-year cumulative OS, CSS, and recurrence-free survival in the RNU group ranged from 43 to 67%, 64–86%, and 22–69%, respectively.

In addition to oncologic outcomes, preservation of renal function has been receiving increased focus in the management of UTUC. Previous reports have shown that patients undergoing nephrectomy or RNU could experience renal insufficiency, with a median relative reduction in eGFR of 21–24%^24,25^. Historically, there has been a misconception that solitary kidney status after RNU does not result in serious morbidities as long as the patient has a normal contralateral kidney. However, changes regarding this view, and in the context of optimal preservation of renal function in the management of UTUC, have led to the use of KSS to minimize renal unit loss without compromising oncologic safety. In this context, and compared with RNU, SU could have more clinical benefits in terms of renal function preservation. Several previous studies showed better preservation of renal function in patients who underwent SU compared to those who underwent RNU^11,13,22^. In agreement with such reports, our results also support the beneficial effect of SU in patients with UTUC in terms of postoperative renal function preservation compared to RNU. In this study, the number of patients with eGFR < 60 mL/min/1.73 m^2^ decreased from 42.5% (17/40) preoperatively to 35.3% (12/34) postoperatively in the SU group, while it increased from 32.5% (13/40) preoperatively to 85.0% (34/40) postoperatively in the SU group. Thus, SU may protect from surgically induced renal insufficiency and reduce the side effects associated with solitary kidney. Furthermore, preservation of renal function could contribute to enhanced tolerance of adjuvant chemotherapy in patients with UTUC who are at high risk of relapse and death from cancer.

Several limitations need to be considered when interpreting our results. First and foremost, our data were derived from a non-randomized and retrospective analysis. Although we conducted propensity score matching to minimize the impact of observable confounders, there is still a risk of selection bias regarding treatment method and follow-up. In this study, we tried to only include ureter tumors in both groups but, due to the low number of patients undergoing SU, the inclusion of renal pelvis tumors in the RNU group after adjustment for propensity score matching was inevitable. That may also have created a selection bias. Second, the number of patients who underwent lymph node dissection was relatively small. However, there is ongoing debate about the therapeutic benefits of lymph node dissection in UTUC and so it has not yet been recognized as part of standard care for UTUC. Based on this, routine lymph node dissection was not performed in this study but only in patients with observable lymphadenopathy on preoperative imaging. Finally, we acknowledge that the small number of patients included in this study and the relatively short follow-up duration may diminish the significance of our findings. Further prospective randomized studies with a longer follow-up are warranted to confirm the benefit of SU on oncologic outcomes and renal function. Despite the aforementioned limitations, this study provides evidence that supports the use of SU as an acceptable alternative to RNU in appropriately selected patients with UTUC.

## Conclusions

Our data showed that the oncologic outcome of SU is comparable to that of RNU in patients with UTUC, even in patients with advanced stage and/or high-grade tumors. Moreover, SU demonstrated a better preservation of renal function compared to RNU. Therefore, SU is a reasonable option for management in appropriately selected patients with UTUC.

## Supplementary Information

Supplementary Figures.
