# Supplementary material for: Comparison of oncologic and functional outcomes between radical nephroureterectomy and segmental ureterectomy for upper urinary tract urothelial carcinoma
Source: Sci Rep. 2021 Apr 9;11:7828. doi: 10.1038/s41598-021-87573-5 (PMC8035162; doi:10.1038/s41598-021-87573-5)
Supplement: Supplementary file 1 — Supplementary Figures. [file 41598_2021_87573_MOESM1_ESM.pdf]

Comparison of oncologic and functional outcomes between radical nephroureterectomy and segmental ureterectomy for upper urinary tract urothelial carcinoma

Tae Heon Kim, MD, PhD[1*], Chung Un Lee. M.D[2*], Minyong Kang, MD, PhD[2], Hwang Gyun Jeon, MD, PhD[2], Byong Chang Jeong, MD, PhD[2], Seong Il Seo, MD, PhD[2], Seong Soo Jeon, MD, PhD[2], Hyun Moo Lee, MD, PhD[2], Hyun Hwan Sung, MD, PhD[2]

[1]Department of Urology, CHA Bundang Medical Center, CHA University, Seongnam, Republic of Korea

[2]Department of Urology, Samsung Medical Center, Sungkyunkwan University School of Medicine, Seoul, Republic of Korea

*Tae Heon Kim and Chung Un Lee contributed equally to this work.

Running head: Segmental ureterectomy for UTUC

Address for correspondence:

Hyun Hwan Sung, M.D., Ph.D.

Department of Urology, Samsung Medical Center, Sungkyunkwan University School of

Medicine, 81, Irwon-ro, Gangnam-gu, Seoul, 06351, Korea

Tel.: +82-2-3410-0737

Fax: +82-2-3410-3027

E-mail address: hhsunguro@gmail.com

Supplementary Materials

Figure S1. In patients with pT2-4, Kaplan–Meier analysis depicting (A) overall survival; (B) cancer-specific survival; (C) progression-free survival; (D) intravesical recurrence-free survival for patients who underwent RNU or SU, after 1:1 propensity score matching. RNU = radical nephroureterectomy; SU = segmental ureterectomy

(A)

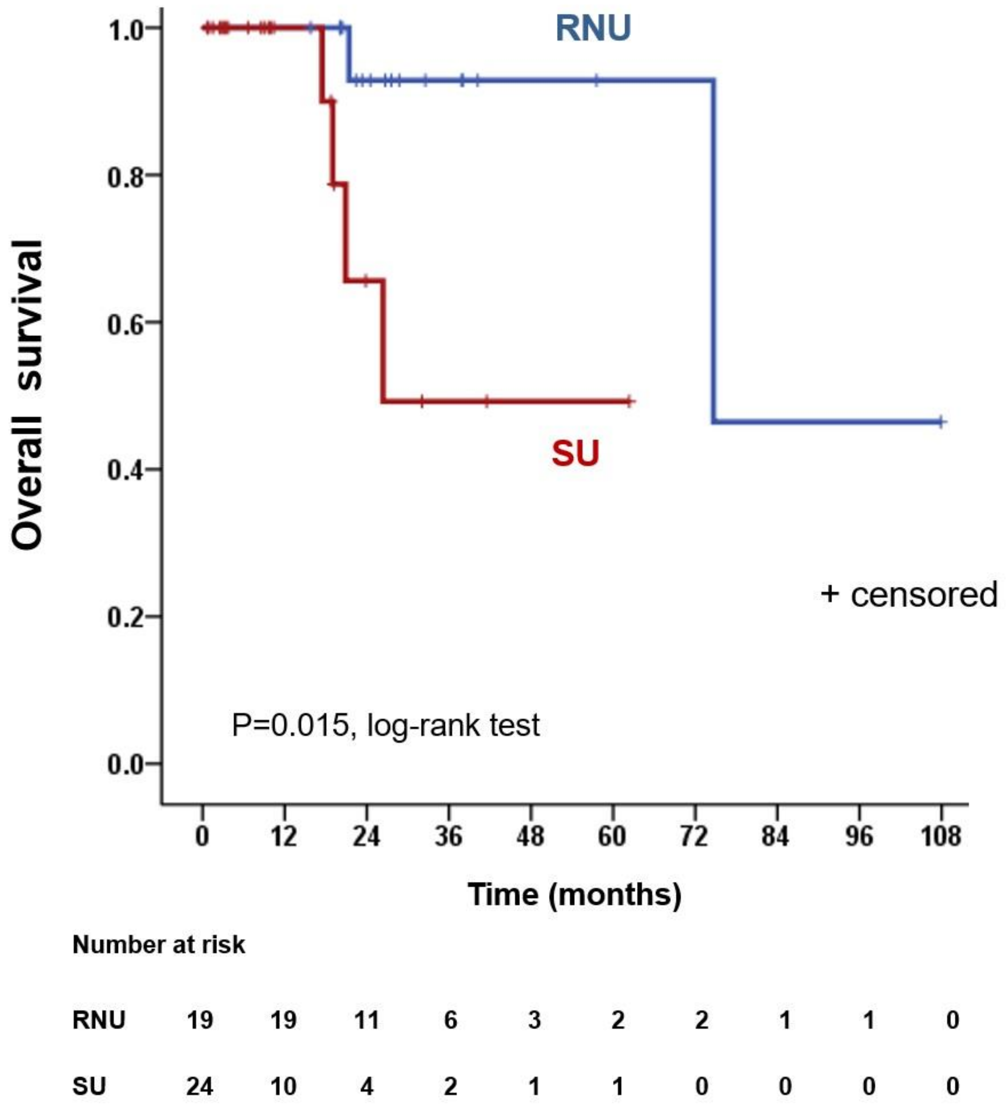

(B)

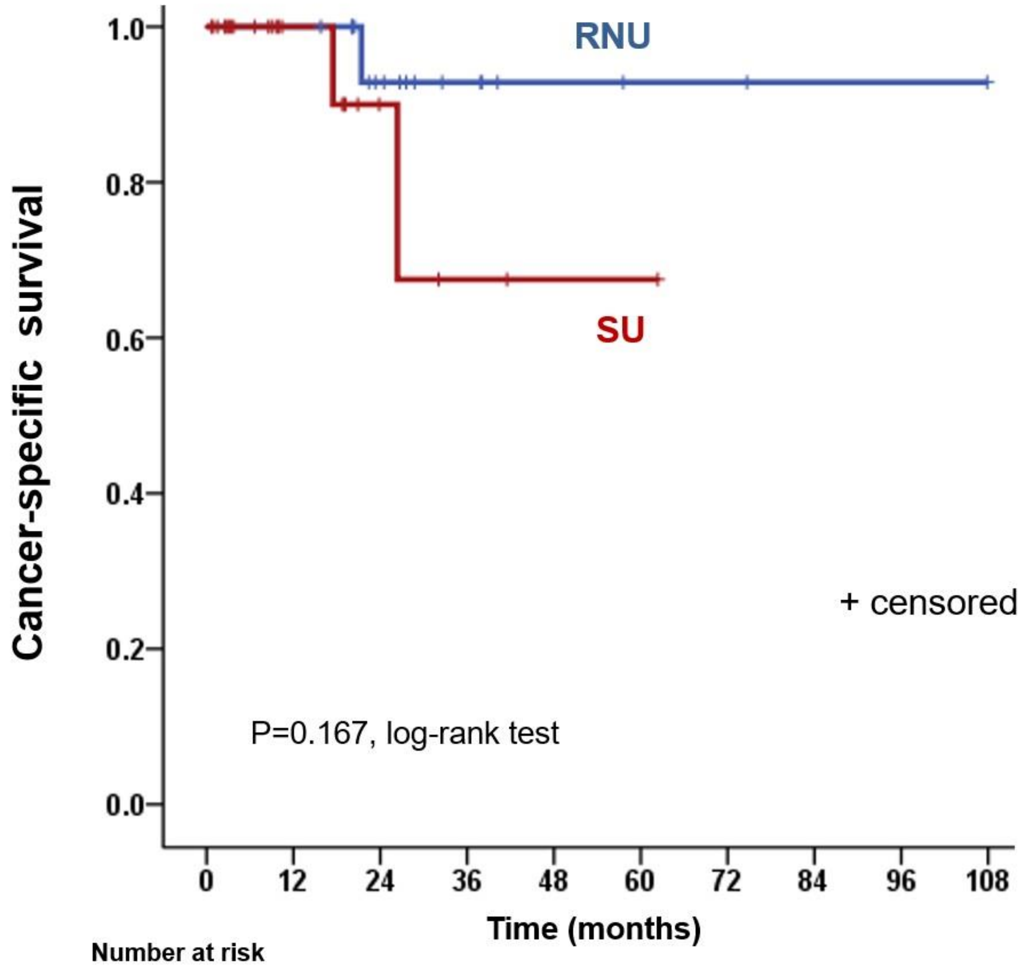

Number at risk

**RNU**	19	19	11	6	3	2	2	1	1	0
**SU**	24	10	4	2	1	1	0	0	0	0

(C)

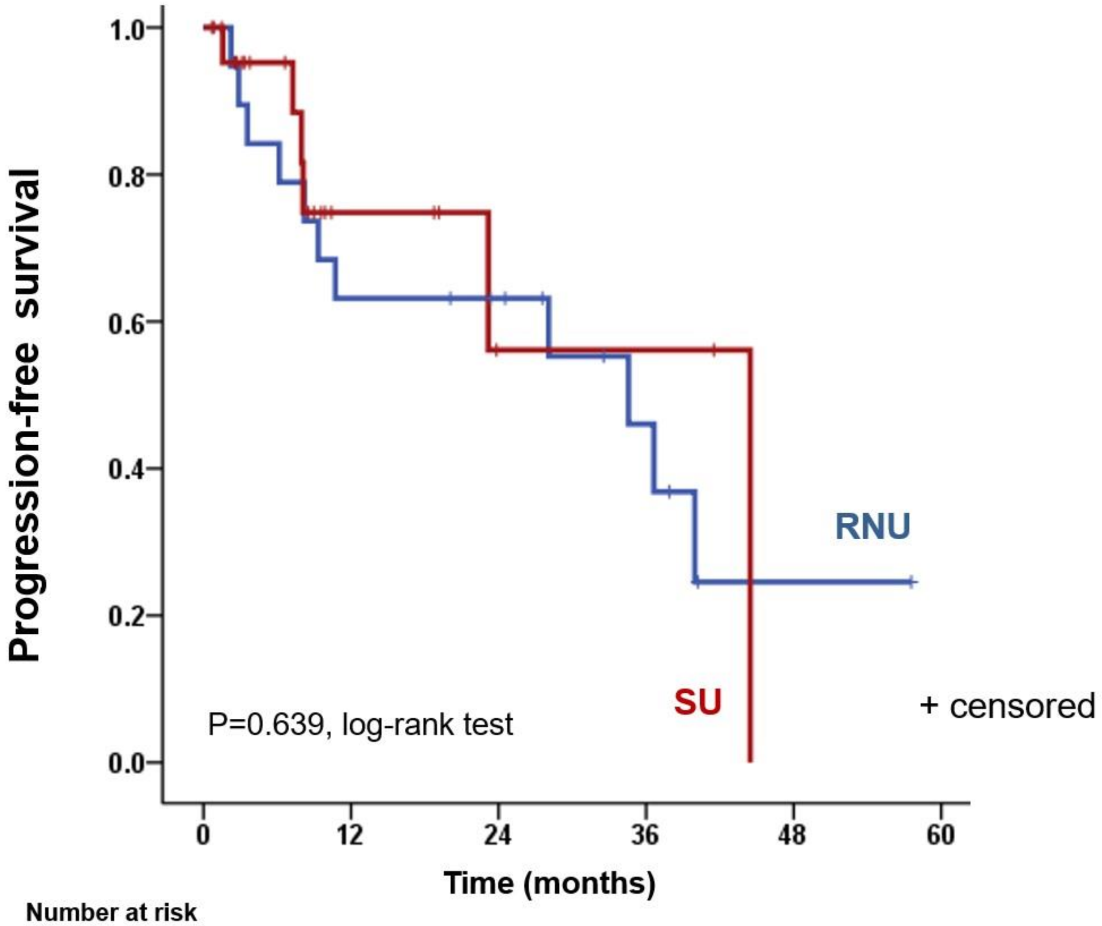

Number at risk

RNU	19	12	10	5	1	0
SU	24	6	2	2	0	0

(D)

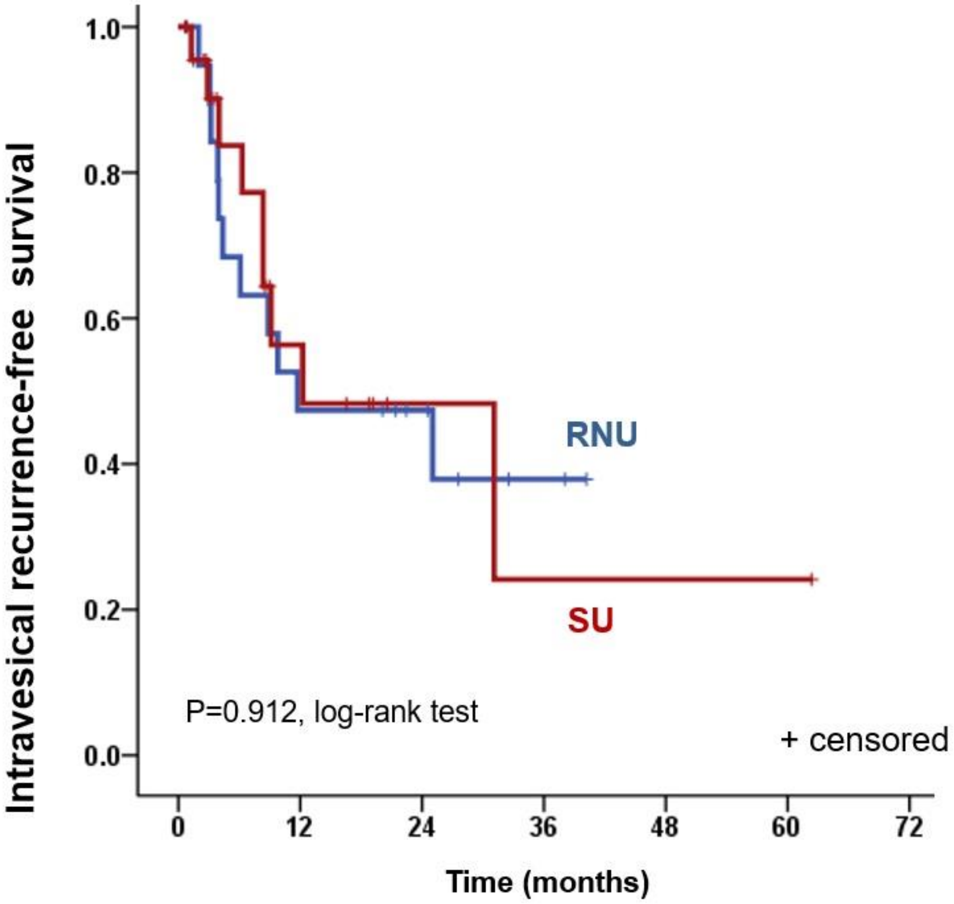

Number at risk

RNU	19	9	6	2	0	0	0
SU	24	7	2	1	1	1	0

Figure S2. In patients with tumor grade 3, Kaplan–Meier analysis depicting (A) overall survival; (B) cancer-specific survival; (C) progression-free survival; (D) intravesical recurrence-free survival for patients who underwent RNU or SU, after 1:1 propensity score matching. RNU = radical nephroureterectomy; SU = segmental ureterectomy

(A)

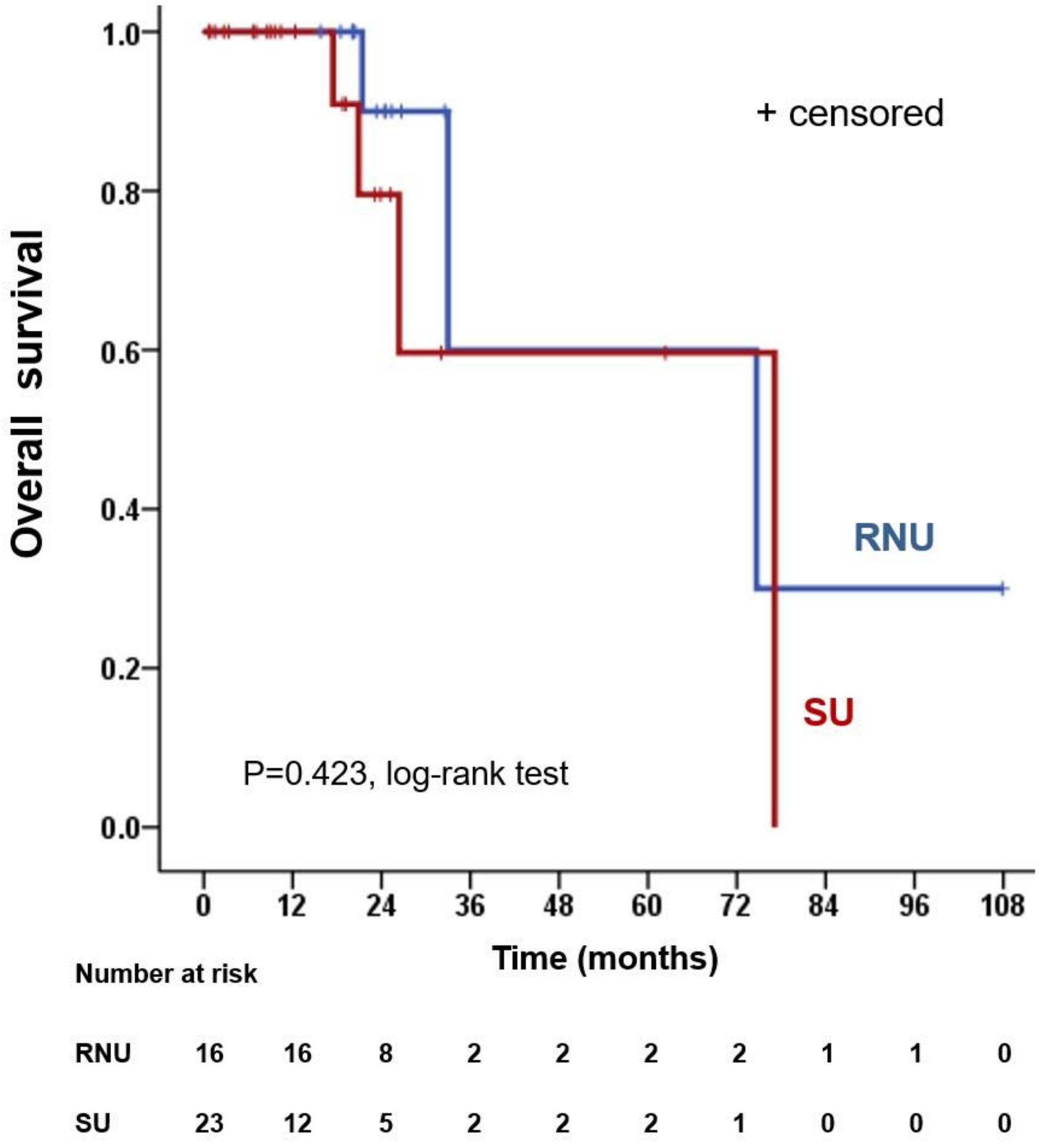

(B)

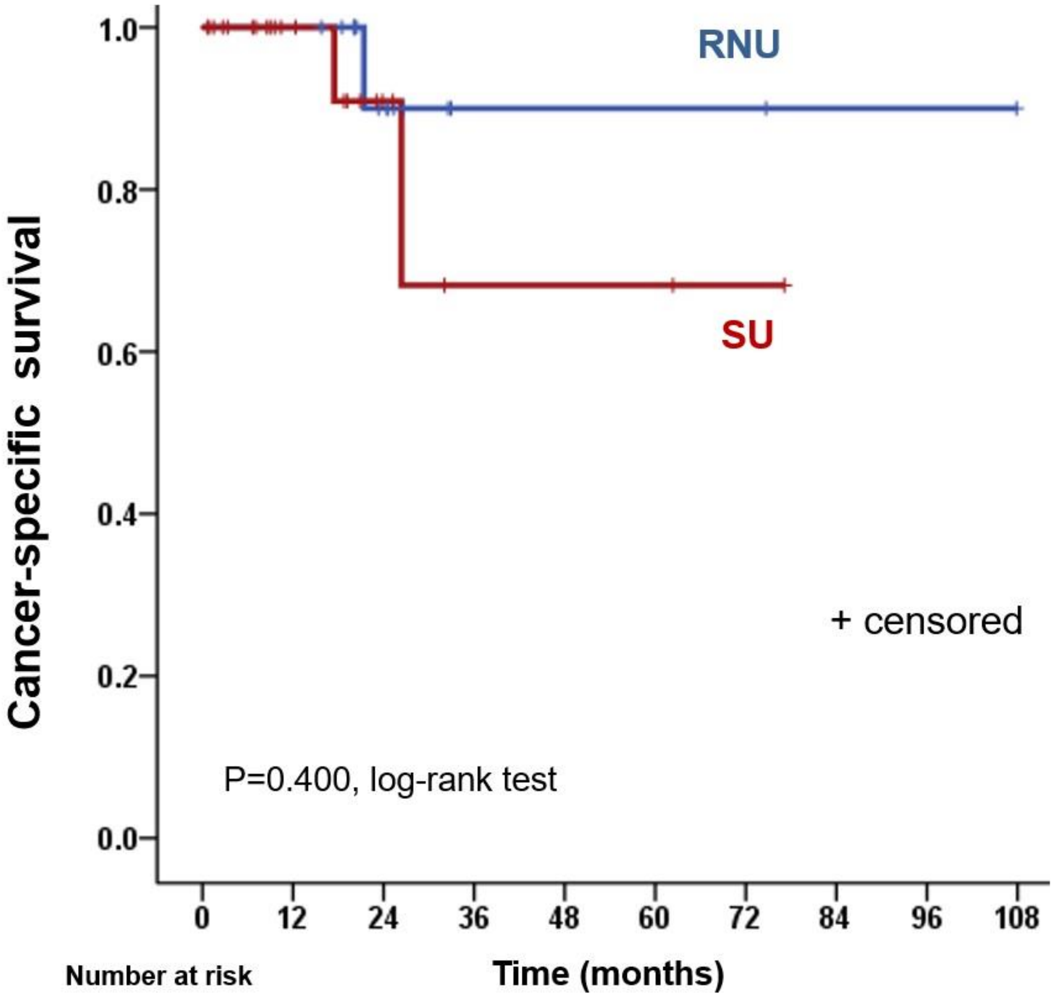

Number at risk

RNU	16	16	8	2	2	2	2	1	1	0
SU	23	12	5	2	2	2	1	0	0	0

(C)

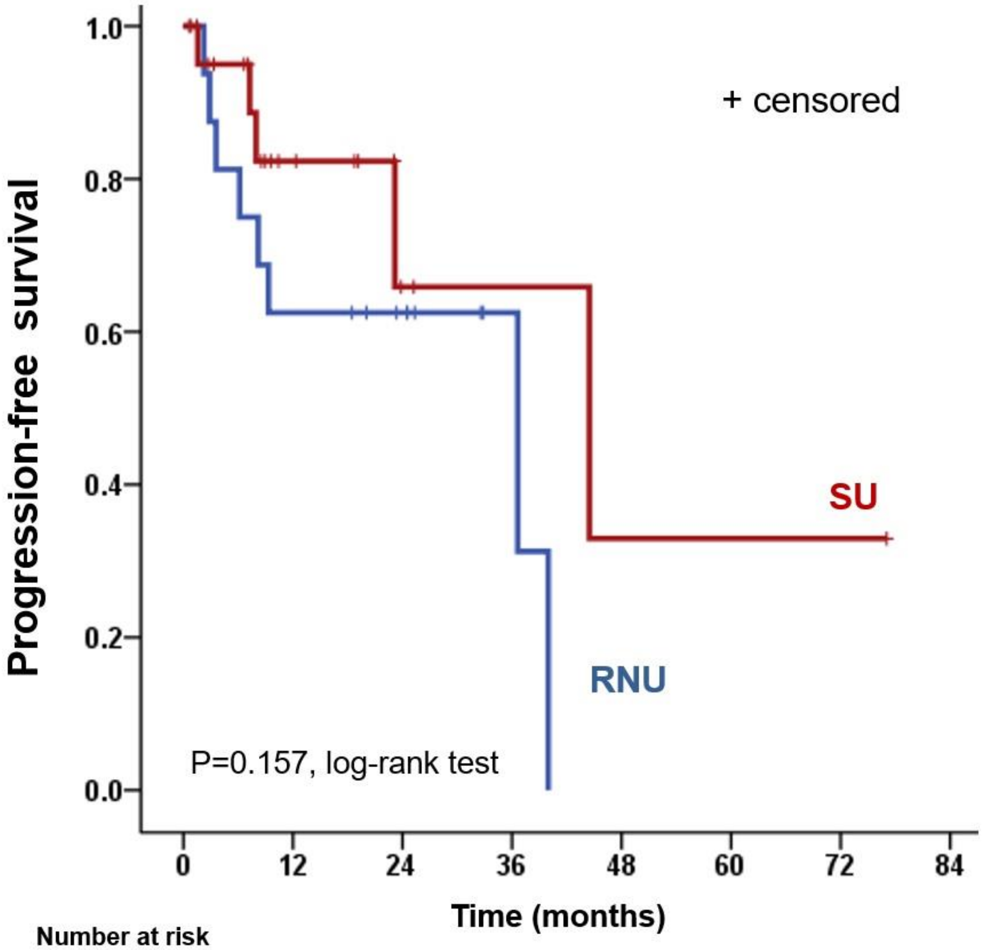

Number at risk

RNU	16	10	7	2	0	0	0	0
SU	23	9	3	2	1	1	1	0

(D)

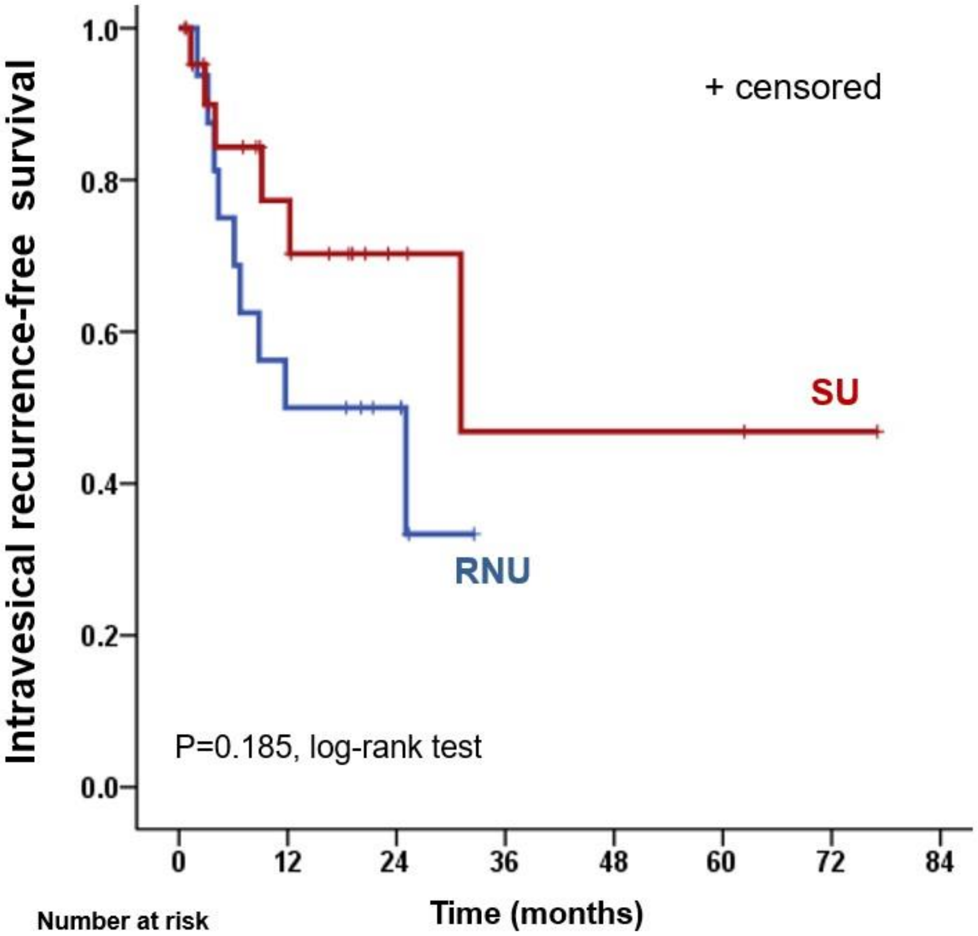

Number at risk

RNU	16	8	5	0	0	0	0	0
SU	23	11	4	2	2	2	1	0
